# Microglial physiological properties and interactions with synapses are altered at presymptomatic stages in a mouse model of Huntington’s disease pathology

**DOI:** 10.1186/s12974-020-01782-9

**Published:** 2020-04-02

**Authors:** Julie C. Savage, Marie-Kim St-Pierre, Micaël Carrier, Hassan El Hajj, Sammy Weiser Novak, Maria Gabriela Sanchez, Francesca Cicchetti, Marie-Ève Tremblay

**Affiliations:** 1 Axe neurosciences, Centre de recherche du CHU de Québec-Université Laval, 2705, Boulevard Laurier, T2-50, Québec, QC G1V 4G2 Canada; 2 Waitt Advanced Biophotonics Core, Salk Institute for Biological Studies, La Jolla, CA USA; 3 Département de psychiatrie et neurosciences, Faculté de médecine, Université Laval, Québec, QC Canada; 4 Département de médecine moléculaire, Faculté de médecine, Université Laval, Québec, QC Canada; 5 Division of Medical Sciences, University of Victoria, Victoria, British Columbia Canada

**Keywords:** Microglia, Huntington’s disease, Electron microscopy, Phagocytosis, Synapses

## Abstract

**Background:**

Huntington’s disease (HD) is a dominantly inherited neurodegenerative disorder that affects cognitive and motor abilities by primarily targeting the striatum and cerebral cortex. HD is caused by a mutation elongating the CAG repeats within the *Huntingtin* gene, resulting in HTT protein misfolding. Although the genetic cause of HD has been established, the specific susceptibility of neurons within various brain structures has remained elusive. Microglia, which are the brain’s resident macrophages, have emerged as important players in neurodegeneration. Nevertheless, few studies have examined their implication in HD.

**Methods:**

To provide novel insights, we investigated the maturation and dysfunction of striatal microglia using the R6/2 mouse model of HD. This transgenic model, which presents with 120+/-5 CAG repeats, displays progressive motor deficits beginning at 6 weeks of age, with full incapacitation by 13 weeks. We studied microglial morphology, phagocytic capacity, and synaptic contacts in the striatum of R6/2 versus wild-type (WT) littermates at 3, 10, and 13 weeks of age, using a combination of light and transmission electron microscopy. We also reconstructed dendrites and determined synaptic density within the striatum of R6/2 and WT littermates, at nanoscale resolution using focused ion beam scanning electron microscopy.

**Results:**

At 3 weeks of age, prior to any known motor deficits, microglia in R6/2 animals displayed a more mature morphological phenotype than WT animals. Microglia from R6/2 mice across all ages also demonstrated increased phagocytosis, as revealed by light microscopy and transmission electron microscopy. Furthermore, microglial processes from 10-week-old R6/2 mice made fewer contacts with synaptic structures than microglial processes in 3-week-old R6/2 mice and age-matched WT littermates. Synaptic density was not affected by genotype at 3 weeks of age but increased with maturation in WT mice. The location of synapses was lastly modified in R6/2 mice compared with WT controls, from targeting dendritic spines to dendritic trunks at both 3 and 10 weeks of age.

**Conclusions:**

These findings suggest that microglia may play an intimate role in synaptic alteration and loss during HD pathogenesis.

## Background

Huntington’s disease (HD) is a dominantly inherited neurodegenerative disorder characterized by loss of motor control, accompanied by cognitive and psychiatric impairments [1]. It is caused by a CAG repeat expansion within exon 1 of the huntingtin (HTT) gene [2], which is ubiquitously expressed across the body and is required for normal development [3, 4]. CAG expansion of 40 or more repeats (compared to healthy individuals’ 6–30) [5] impairs the protein’s folding, eventually resulting in intracellular inclusions of mutant huntingtin (mHTT) across the brain [6]. Patients usually begin to manifest symptoms between 35 and 45 years of age, but the length of the CAG expansion is inversely correlated with age of disease onset, and individuals with more than 50 CAG repeats present with symptoms before age 20 [7]. Several animal models, including the R6/2 mouse, have been generated to study the effects of mHTT in the brain [8]. These animals express exon 1 of human HTT with ~ 120–150 CAG repeats under its endogenous promoter. Disease onset in these animals is between 6 and 9 weeks of age and animals generally die between 12 and 14 weeks of age [8].

Medium-sized spiny neurons (MSSNs) make up 95% of the neurons within the striatum and are particularly vulnerable to CAG repeat expansion [9, 10]. They are among the first neurons to die within the striatum of HD patients [9], though by later disease stages there is widespread loss of pyramidal neurons in the cerebral cortex as well [11]. Striatal neurodegeneration is observable in HD patients and moves along a dorsal-to-ventral and medial-to-lateral pattern [12]. Although the genetic cause of HD has been determined, the specific susceptibility of MSSNs has not been fully explained. Electron dense, dark neurons containing condensed cytoplasm and other markers of cellular stress have been identified using transmission electron microscopy (TEM) in postmortem brains of HD patients as well as in late-stage (17-week-old) R6/2 mice [13]. Furthermore, reductions in synaptophysin and postsynaptic density 95 staining were measured in the striatum and cerebral cortex of late-stage R6/2 mice between 10 and 12 weeks of age [14, 15]. In fact, reductions in synaptic markers within the somatosensory cortex are seen as early as 6 weeks of age in R6/2 mice, just before the onset of behavioral impairments [16].

There have been relatively few studies on the involvement of microglial cells in HD. In fact, mHTT inclusions have been found in all cell types of the brain in both mouse models and human cases of HD [17], while cell-type specific expression of mHTT in glial cells, either oligodendrocytes or astrocytes, is sufficient to cause motor deficits and results in early death across several mouse models [18, 19]. In addition to studies that focused on oligodendrocytes and astrocytes, a relatively small amount of attention has been paid to the involvement of microglia, the brain’s resident macrophages, in HD pathogenesis. Microglia are responsible for normal synaptic pruning and maintenance and have been implicated in a number of disease states associated with synaptic loss and neurodegeneration [20, 21]. Recently, microglia have begun to be studied in the specific context of HD [22]. Morphologically reactive microglia (defined by large, amoeboid-like cell bodies with short or absent processes) have been identified in postmortem samples of cerebral cortex and striatum from HD patients, as well as in the striatum of mouse models of HD [23, 24].

In mice, microglia from wild-type (WT) animals co-cultured with striatal neurons expressing mHTT depicted increased proliferation, elevated levels of cytokine IL-6 and complement components C1qa and C1qb, and took on a more amoeboid morphology. In spite of their reactive phenotype, the presence of microglia within the culture increased mHTT neuronal viability [25]. Interestingly, microglia, macrophages, and monocytes isolated from human mHTT carriers or from the YAC128 mouse model also expressed elevated levels of IL-6 and other proinflammatory markers in response to the proinflammatory stimulus lipopolysaccharide, as measured by multiplex ELISA [26]. More recent work determined that the aberrant reactivity of microglia in HD may be cell-autonomous, considering that mHTT expression within these cells led to increased expression of transcription factors PU.1 and CEBP which are responsible for macrophage and microglia development as well as maturation [27]. Increases in PU.1 and CEBP in mHTT-expressing microglia resulted in microglial “priming” or enhancement of proinflammatory gene expression, including IL-6 and TNFα, driven downstream of NFκB activation [27].

In human cases of HD, positron emission tomography (PET) imaging has identified increased microglial reactivity in the striatum and cortical regions of symptomatic HTT patients together with brain-wide increases of radiotracer binding in presymptomatic HTT carriers [28–30]. In all cases, ^11^C-(R)-PK11195 was used as a translocator protein (TSPO) binding to mitochondrial peripheral benzodiazepine sites upregulated in microglia and other mononuclear phagocytes in response to proinflammatory stimuli or in neurodegenerative conditions such as Alzheimer’s disease [31]. In two cases, increased microglial reactivity was correlated with decreased dopaminergic signaling, as read by D2 receptor binding with ^11^C-raclopride-PET ligand in the identified regions [28, 29]. In addition, postmortem studies of human tissue have identified increases in complement components, including C1q, C3, C4, iC3b, and C9, in HD patients [32]. Together, these data suggest that primed microglia observed in presymptomatic patients may react to normal stimuli in a hyperactive fashion, worsening disease pathogenesis.

While there have been several recent studies investigating the potential role of microglia in HD, the literature has focused on the inflammatory function of these brain-resident immune cells. In addition to their neuroinflammatory roles in disease, microglia are now considered to exert beneficial physiological roles, notably in synaptic pruning and maintenance during development and adulthood [33]. It has been hypothesized that early HD symptoms may be a result of loss of synaptic input onto the MSSNs in the striatum [34]. In order to investigate the role that microglia might play in the synaptic loss seen in HD, we have performed light microscopy studies to uncover densitometric, morphological, and phagocytic alterations in microglia among the striatum of sex- and age-matched WT and littermate R6/2 mice. We investigated 3-week-old mice prior to neuronal loss or the manifestation of motor phenotypes, as well as 10-week-old mice when motor impairments were quantifiable, and 13-week-old mice when motor phenotypes were severe.

Here, we present light microscopy data which demonstrate that mHTT microglia are morphologically more mature earlier than WT microglia at 3 weeks of age and are hyperphagocytic at early ages. These early microglial alterations were present even before disease-related signs unfold in the R6/2 model. Bolstered by this information, we delved further into the phagocytic alterations and investigated 3-week-old and 10-week-old animals using quantitative transmission electron microscopy (TEM). Microglial ultrastructure was already altered in the dorsomedial striatum of 3-week-old animals. Furthermore, mHTT microglia were found to interact differently with synapses: they were more likely to contact synaptic clefts prior to synaptic loss and less likely to contact synaptic clefts after motor symptoms were displayed. Finally, we utilized state-of-the-art focused ion beam scanning electron microscopy (FIB-SEM) to investigate in 3-dimension (3D) synaptic structures in 3-week-old and 10-week-old R6/2 versus WT animals. While total synaptic input onto dendrites was not reduced in 3-week-old animals, we found that synaptic inputs onto dendrites in the dorsomedial striatum of R6/2 animals were more likely to make en face synapses, targeting dendritic trunks instead of dendritic spines. This occurred before synaptic loss and persisted throughout disease pathology, concurrent with altered microglia-synaptic interactions. Together, these data suggest that mHTT microglia may be implicated in HD disease development and/or progression. Further studies are required to clarify this potentially important role.

## Materials and methods

### Animals

Sex- and age-matched R6/2 B6CBA (120+/-5 CAG) and nontransgenic littermate mice on a mixed C57BL/6/CBA background were purchased from The Jackson Laboratory and group-housed 3 to 5 animals per cage until sacrifice. Animals were kept under a 12-h light/dark cycle with food and water provided ad libitum. All the experiments were approved and performed under the guidelines of the Institutional animal ethics committees, in conformity with the Canadian Council on Animal Care recommendations.

### Tissue collection

Three-, 10-, and 13-week-old R6/2 mice or nontransgenic littermates were anesthetized with 80 mg/kg sodium pentobarbital (i.p. injection) prior to transcardiac perfusion. Prior to anesthetization, hindlimb clasping was verified in all 10- and 13-week-old R6/2 mice [35]. Animals were perfused through the aortic arch with 3.5% acrolein followed by 4% paraformaldehyde (PFA) for electron microscopy (EM), or solely with 4% PFA for light microscopy [36]. Brains collected for light microscopy were post-fixed for 48 h in 4% PFA and dehydrated in 15% and 30% sucrose solutions before coronal sections (40-μm thick) were cut using a freezing microtome [35]. For EM, brains were extracted and post-fixed for 90 min in 4% PFA before coronal sections (50-μm thick) were cut in phosphate-buffered saline (PBS, 50 mM, pH 7.4) using a Leica VT1000s vibratome [37]. Brain sections for both light and EM were collected and stored in cryoprotectant at − 20 °C. Brain sections from Bregma levels 0.5 mm to 0.7 mm were selected based on the stereotaxic atlas of Paxinos and Franklin (4th edition) and examined for light, TEM, or FIB-SEM experiments.

### Immunohistochemistry

Immunoperoxidase staining for light microscopy and TEM (immunoEM) was performed as described previously [37]. Briefly, brain sections were washed in PBS, quenched 5 min with hydrogen peroxide (2% for light microscopy, 0.3% for immunoEM), followed by a 30-min incubation in 0.1% sodium borohydrate (NaBH_4_). Sections were washed in Tris-buffered saline (TBS; 50 mM at pH 7.4) containing 0.01% Triton X100, and processed freely floating for immunostaining. Sections were blocked for 1 h in TBS containing 10% fetal bovine serum, 3% bovine serum albumin, and Triton X100 (1% for light microscopy, 0.1% for immunoEM), prior to overnight incubation at 4 °C with primary antibody against ionized calcium binding adaptor molecule 1 (IBA1) in blocking buffer (rabbit anti-IBA1 1:1000, Wako). Sections were then washed and incubated with secondary antibody (biotin-conjugated goat anti-rabbit IgG 1:200, Jackson Immunoresearch) for 1.5 h. Staining was amplified with the ABC Vectastain system (1:100, Vector Laboratories) and revealed using diaminobenzidine (DAB; 0.05%) and hydrogen peroxide (0.015%) in Tris buffer (TB; 0.05 M at pH 8) for 5 min. Sections were mounted onto slides and coverslipped for light microscopy or post-fixed flat for 30 min in 1% osmium tetroxide and further processed for EM as described below.

For FIB-SEM, sections were post-fixed flat in 2% osmium tetroxide and 1.5% potassium ferrocyanide for 1 h, followed by incubation in 1% thiocarbohydrazide for 20 min, and further incubated in 2% osmium tetroxide as described by the National Center for Microscopy and Imaging Research [38]. Following post-fixation, tissues for both TEM and FIB-SEM were dehydrated using increasing concentrations of ethanol and finally immersed in propylene oxide. Following dehydration, sections were impregnated with Durcupan resin (Electron Microscopy Sciences; EMS) overnight at room temperature, mounted between ACLAR embedding films (EMS) and cured at 55 °C for 72 h. Specific regions of interest (1 mm × 1 mm square of dorsomedial region of the striatum) were excised and mounted on resin blocks for ultrathin sectioning.

Immunofluorescent staining for light microscopy was performed as described previously with minor modifications to adapt the protocol for free-floating sections [39]. Briefly, free-floating sections were washed in PBS, incubated in 0.1 M citrate buffer for 15 min at 90 °C, washed, and incubated for 1 h using block of 2% normal donkey serum in PBS containing 0.2% Triton X-100. Sections were incubated with primary antibodies (rabbit anti-IBA1, 1:1000, Wako; rat anti-CD68 1:2500; BioRad) in blocking buffer overnight at 4 °C. After primary antibody incubation, sections were washed and incubated with fluorescent secondary antibodies (donkey anti-rabbit alexa fluor 546, donkey anti-rat alexa fluor 488 1:1000, Invitrogen). Following staining, sections were counterstained with 4′,6-diamidino-2-phenylindole (DAPI, Thermo Fisher, 200 nM in PBS) and mounted on glass slides using Fluoromount G (Thermo Fisher).

### Microglial imaging and analysis

#### Light microscopy

IBA1-immunoreactive (+) cells from the dorsomedial, ventromedial, dorsolateral, and ventrolateral regions of the striatum were imaged using a Nikon eclipse TE300 light microscope (DAB staining) or confocal fluorescent microscope (IBA1 and CD68 double staining). Microglial density, distribution, morphology, and CD68+ puncta were analyzed as described previously by researchers blinded to animal age and genotype using ImageJ [39–41]. Microglial density within the striatum was measured across 4 sections per animal imaged at × 4 magnification by marking the center of each IBA1+ microglial cell with a dot using the paintbrush tool. The “analyze particles” function was used to count cell numbers and use spatial coordinates to determine the nearest neighbor distance (NND), while microglial density was determined by dividing the number of cells by the total surface area of the regions of interest measured in square millimeters for each animal. The spacing index was calculated as the square of the average NND multiplied by the microglial density per animal. Microglial morphology studies were performed on 40 cells per animal imaged at × 40 magnification. Cell body size was determined by encircling the microglial soma using the freehand selection tool, and arborization area was measured using the polygon tool to connect distal extremities of every process and reported in micrometers. The morphological index was calculated by dividing the soma area by the arborization area for each cell. Density of CD68+ puncta within IBA1+ microglial cells (number of puncta per cell body) was calculated within the dorsomedial region of the striatum. Between 20 and 27 cells across 5 sections per animal (total 60–100 cells per condition) were imaged at × 63 using a Zeiss LSM800 confocal microscope. Regions of interest were drawn around IBA1+ cell bodies as described [42], and the number of CD68+ puncta was counted.

#### TEM

Ultrathin (65–80 nm) sections were cut with a diamond knife (Diatome) on a Leica UC7 ultramicrotome, collected on bare square mesh grids (EMS), and imaged at 80 kV with a FEI Tecnai Spirit G2 transmission electron microscope. Profiles of neurons, synaptic elements, and microglia were identified according to well-established criteria [43]. Microglia were identified both by their IBA1 immunoreactivity as well as their association with extracellular space pockets, distinctive long stretches of endoplasmic reticulum (ER), and small elongated nucleus [37]. Between 7 and 11 microglial cell bodies (imaged using magnifications between × 4800 and × 9300) and 70 to 100 microglial cell processes (imaged at × 9300) profiles per animal were photographed using an ORCA-HR digital camera and analyzed by blinded researchers using ImageJ, as previously described [39, 44]. Microglial processes were traced using the freehand selection tool and analyzed for their area and perimeter in ImageJ. Contacts with synaptic elements (presynaptic axon terminals identified by their synaptic vesicles, postsynaptic dendritic spines identified by their postsynaptic density, and synaptic clefts identified by the direct apposition with less than 20-nm extracellular space between presynaptic terminals and dendritic spines) were measured by counting direct contacts with microglial plasma membrane. Phagocytic activity was measured as the proportion of microglial cell bodies or processes profiles containing phagosomes, defined as the presence of endosomes containing digested elements or fully lucent vacuoles larger than 300 nm [44]. The total number of phagosomes per microglial cell body or process profile was determined. Dilated ER identified by gaps between cisternae membranes larger than approximately 100 nm was counted, and the proportion of microglial cell body profiles containing dilated ER was reported [44].

#### Focused-ion beam scanning electron microscopy (FIB-SEM)

A Leica UC7 ultramicrotome equipped with a glass knife was used to trim the tissue into a roughly cubic frustum. A diamond knife (Diatome) was used to polish the surface of the tissue and to collect semithin sections that were used to identify a region of interest (ROI) within the block face. The trimmed tissue block was removed from the resin blank using a jeweler’s blade and mounted on an aluminum stub (EMS) using conductive carbon paint (EMS) with the smooth surface facing up [45]. Finally, the sample was sputtered with 30 nm of platinum using a sputter coater (Zeiss). The sample was loaded into a Zeiss Crossbeam 540 FIB-SEM. Once in the FIB-SEM, the region of interest was identified for iterative FIB-milling and SEM imaging. ATLAS Engine 5 software (Fibics) was used to automate the steps involved in FIB-SEM data collection, including the deposition of a protective platinum surface followed by the sequential milling and deposition of a “wolverine claw” of fiducial markers to allow for precise automated focus and drift correction. Images were acquired with 5-nm pixels in the lateral dimensions using the ESB and SE2 detectors with the SEM voltage set at 1.4 kV and current of 1.2 nA. FIB milling steps of 10 nm were accomplished using a milling voltage of 30 kV and current of 1.5 nA. Volumes of 125 μm^3^ in dimension were captured over 18–24-h imaging sessions. To maximize dendritic segments within the volumes, regions of the dorsomedial striatum outside of striosomes and devoid of blood vessels, myelinated axons, and cell bodies were selected. After acquisition, image stacks were evaluated for quality and finely aligned using ATLAS software and were exported as tiff stacks. The carving module of Ilastik software [46] was used for the semi-automated segmentation of 3–6 dendrites and their spines per animal (165 synapses from WT and 189 synapses from R6/2 animals were analyzed). The number and type (onto dendritic spine versus en face synapse directly onto dendritic trunk) of synapses were recorded manually. By segmenting individual dendrites in serial images, we were able to count exact synapse density, instead of estimations used by other synapse counting methods [47]. We segmented and analyzed 280 dendritic spines and 74 synapses directly onto dendritic trunks, similar in number to those analyzed by other 3D EM studies investigating synaptic structures [48]. Supplemental Video 1 was generated from Ilastik-assisted semi-automated segmentations of the FIB-SEM data and proofread with VAST Lite [49]. The segmented objects (a dendrite and two presynaptic axons) were exported from VAST Lite as a series of .png traces and imported into ImageJ as a stack. The traces for individual objects were binarized and converted to meshes using the 3D Viewer in ImageJ [50] before being exported as .stl mesh objects. The .stl mesh models of the dendrite and axons were all imported into Blender [51]. The scene with the models superimposed on the image stack was composed using Neuromorph [52] and Blender and rendered using Blender. All imaging, segmentation, and synaptic counts were performed by an observer blinded to the animal genotype.

### Statistical analysis

The software Prism (GraphPad, version 8) was used to analyze all the acquired data. Two-way ANOVA with Sidak post hoc for multiple comparisons was performed for light and EM experiments. *p* < 0.05 was considered statistically significant. All reported data on graphs represent mean ± standard error of the mean (SEM). For microglial density, distribution, and morphology studies *N* = animal, for phagocytosis and EM studies *N* = cell, process, or dendrite [39, 44].

## Results

### Microglial morphological maturation is accelerated in the R6/2 mouse model

**Fig. 1:**
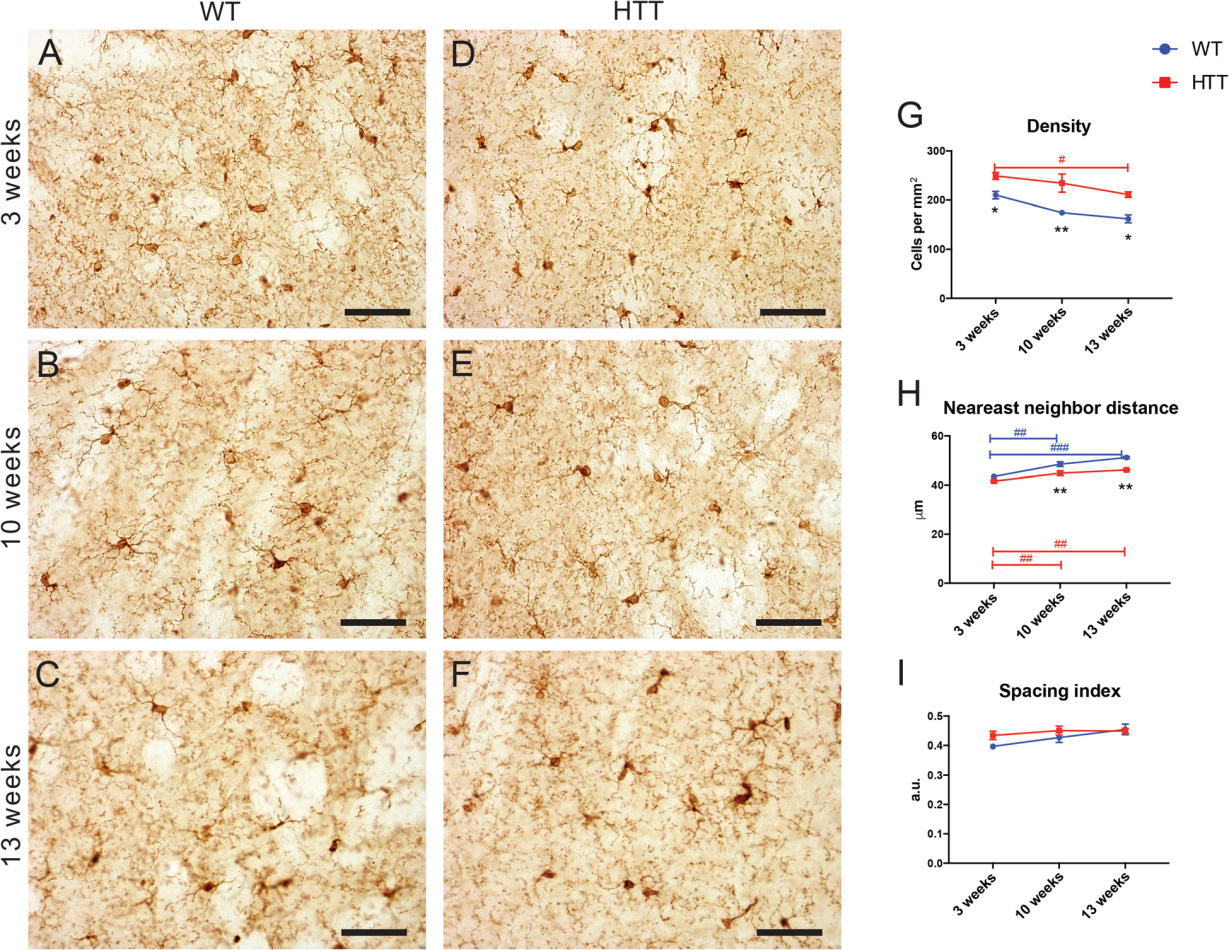
Microglial densitometric changes observed in the striatum over the course of HD pathology. Picture examples showing microglial density and distribution in wild-type (WT) mice at 3 weeks (**a**), 10 weeks (**b**), and 13 weeks (**c**) of age and R6/2 (HTT) mice at 3 weeks (**d**), 10 weeks (**e**), and 13 weeks (**f**) of age, as identified using anti-IBA1 staining, captured using a brightfield microscope. Scale bar = 10 μm. Quantitative analysis of microglial density (**g**), nearest neighbor distance (**h**), and spacing index (**i**) performed at all three ages in both WT and HTT conditions. *N* = 4 animals per condition. Asterisk denotes the difference from WT, blue number sign denotes the difference between ages in WT mice, and red number sign denotes the difference between ages in R6/2 mice; *^,#^*p* < 0.05, **^,##^*p* < 0.01, ^###^*p* < 0.001

In order to investigate microglial maturation and function in the R6/2 mouse model of HD, we performed IBA1 immunostaining followed by densitometric analysis in the striatum of 3-week-, 10-week-, and 13-week-old animals (Fig. 1a–f). Densitometric studies included measurement of density (cells/mm^2^) as well as the nearest neighbor distance (NND, nearest cell to every other cell), and spacing index (compiling the NND and density) between microglia. R6/2 animals displayed an age-dependent decrease in microglial density but had higher microglial density compared to control animals at all ages investigated (Fig. 1g). The NND of both R6/2 and control animals increased with age, and WT animals had significantly larger NND compared with R6/2 animals at both 10 and 13 weeks (Fig. 1h). However, the spacing index did not differ significantly between groups at any age (Fig. 1i).

**Fig. 2:**
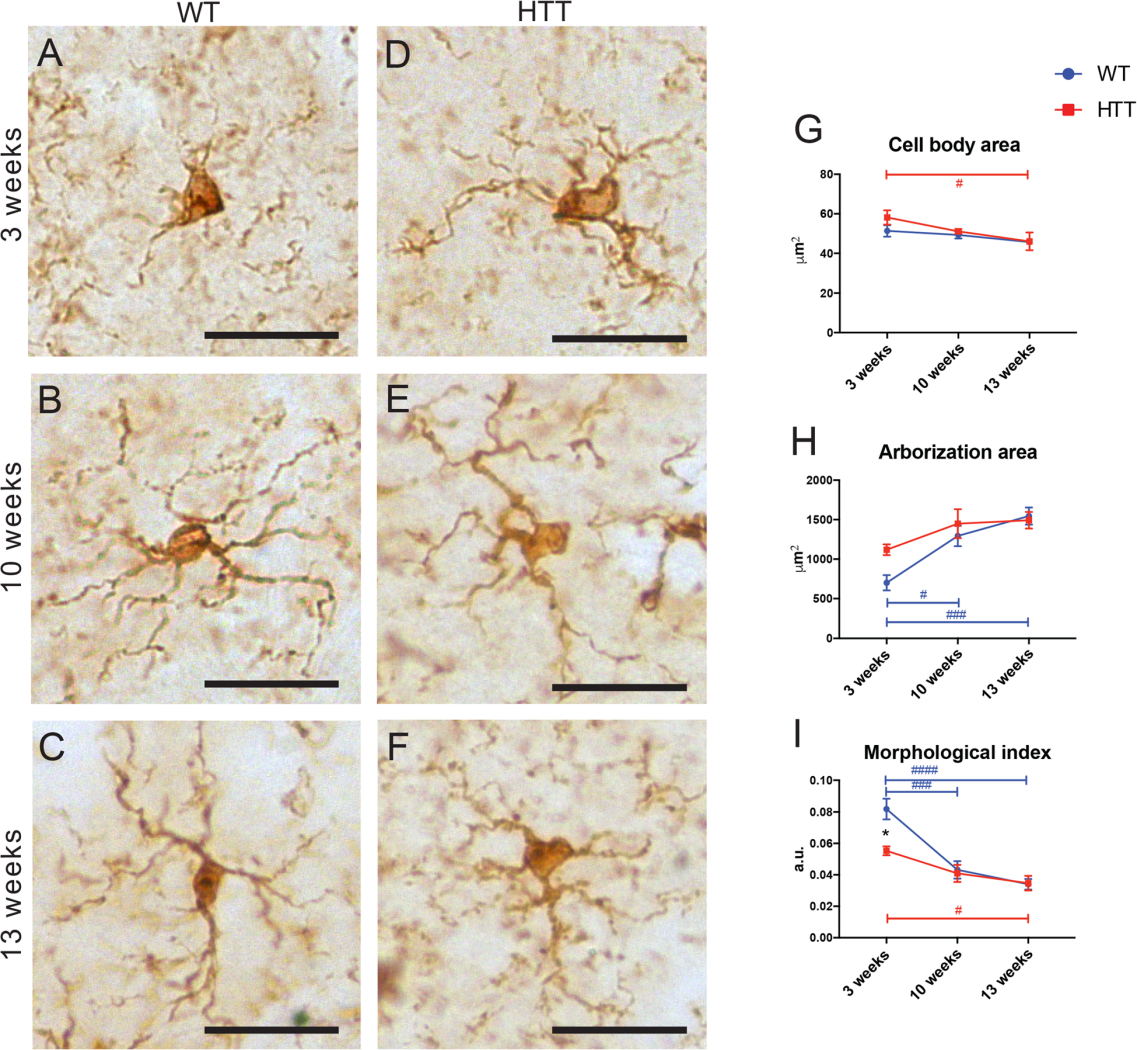
Microglial morphometric changes observed in the striatum over the course of HD pathology. Picture example showing microglial morphology captured by brightfield microscopy in WT mice at 3 weeks (**a**), 10 weeks (**b**), and 12 weeks (**c**) of age and R6/2 (HTT) mice at 3 weeks (**d**), 10 weeks (**e**), and 13 weeks (**f**) of age, identified using anti-IBA1 staining. Scale bar = 5 μm. Quantitative analysis of microglial cell body area (**g**), arborization area (**h**), and morphological index (**i**) performed at all three ages in both WT and HTT conditions. *N* = 4 animals per condition. Asterisk denotes the difference from WT, blue number sign denotes the difference between ages in WT mice, red number sign denotes the difference between ages in R6/2 mice; *^,#^*p* < 0.05, ^###^*p* < 0.001, ^####^*p* < 0.0001

In addition to these changes in densitometric maturation, microglia in both genotypes exhibited an age-dependent morphological maturation. We performed morphometric analysis in the striatum of 3-week-, 10-week-, and 13-week-old animals (Fig. 2a–f). Microglial cell body area was stable across ages in WT animals, while cell body area decreased with age in R6/2 animals (Fig. 2g). Further morphological investigation found microglia in control animals displayed an increase in their arborization area (Fig. 2h) while R6/2 animals’ microglial arborization area was already large at 3 weeks. Both genotypes displayed an age-dependent decrease in their morphological index (the ratio of cell body area over arborization area) (Fig. 2i). In fact, R6/2 mouse microglia displayed a significantly reduced morphological index compared with WT microglia at 3 weeks of age (Fig. 2i). Together, these data indicate that microglial number remained elevated in the striatum of R6/2 mice compared with WT controls and their morphology was significantly altered as early as 3 weeks. This age corresponds to a time point preceding any known inflammatory signaling or neurodegeneration in this model [53].

### Microglial phagocytosis is increased in the R6/2 mouse model

**Fig. 3:**
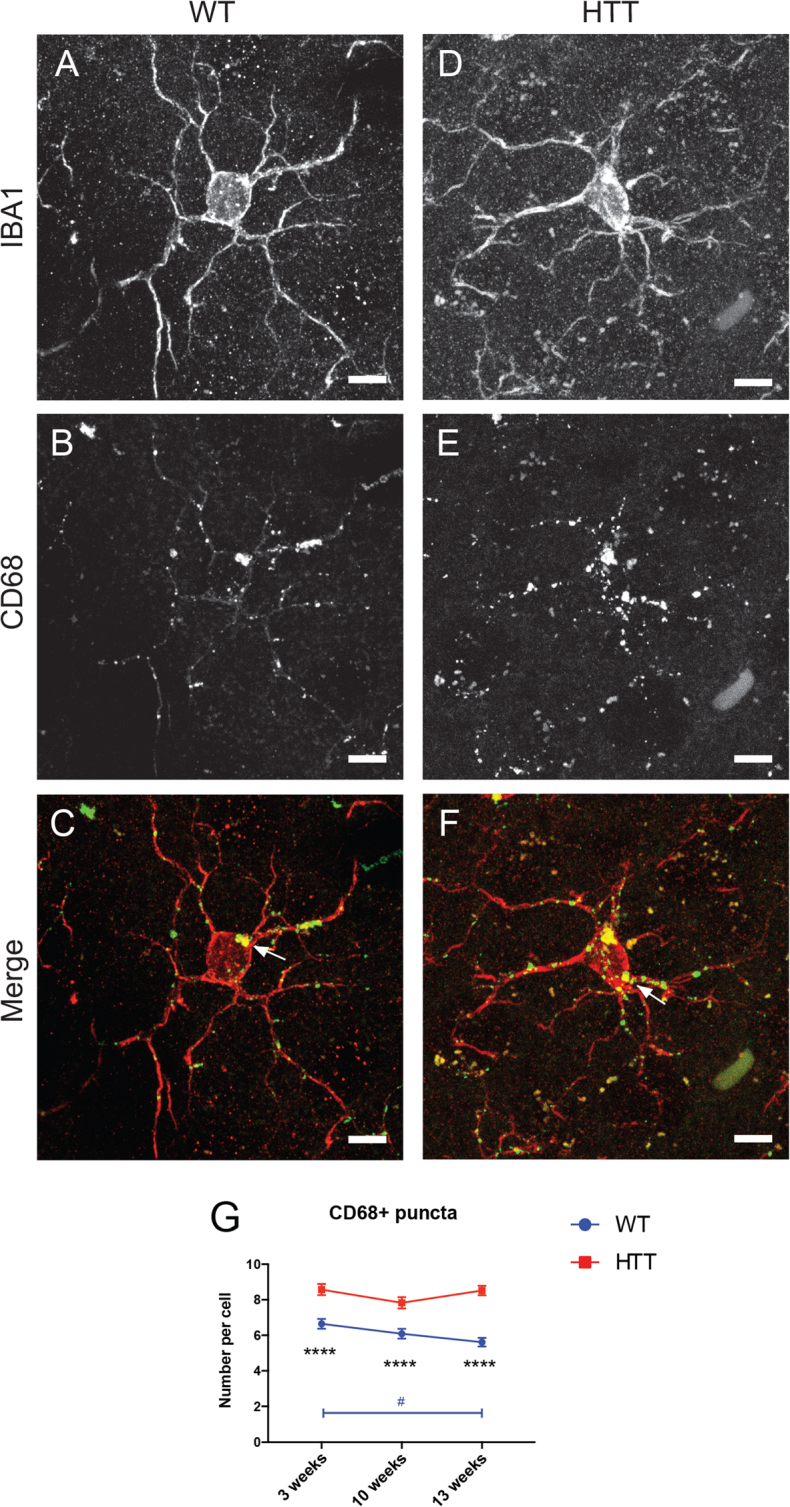
Microglial phagocytosis increases in the striatum over the course of HD pathology. Picture examples of 13-week-old WT (**a**–**c**) and R6/2 (**d**–**f**, HTT) mouse microglia costained with IBA1 (**a**, **d**) and the phagolysosomal marker CD68 (**b**, **e**). Merged images display phagolysosomes (white arrows) within the microglial cell bodies (**c**, **f**). Scale bar = 5 μm. Statistical analysis (**g**) of the number of CD68+ puncta per microglial cell body. *n* = 20–27 cells per animal for all conditions, and data was collected from *N* = 3–4 animals per condition. Asterisk denotes the difference from WT, blue number sign denotes the difference between ages in WT mice; ^#^*p* < 0.05, ****p < 0.0001

Microglia are known to play a major role in synaptic removal and plasticity, notably via phagocytosis in healthy and disease states [20, 54]. To determine the functional implications of the decreased morphological indices in R6/2 mice, we quantified the immunolabeling for CD68, a transmembrane protein highly expressed by microglia and macrophages that is enriched in their phagolysosomal compartments [55]. IBA1+ microglia in both R6/2 and WT mice displayed abundant CD68+ puncta (Fig. 3a–f). We quantified the number of CD68+ puncta per microglial cell body (phagocytic index) in the striatum of 3-, 10-, and 13-week-old animals. WT microglia decreased their phagocytic index as the animals matured (Fig. 3g). However, microglia in R6/2 mice had elevated levels of CD68+ puncta at all ages and did not display a decrease in phagocytosis over time with maturation (Fig. 3g). Microglia in R6/2 mice may be performing aberrant excess phagocytosis as often seen in neurodegenerative disease conditions [20], or their phagolysosomal system may be overwhelmed and not processing phagocytosed material properly, causing the cells to become overloaded with phagocytic debris.

### Microglial cell body ultrastructure is altered in the R6/2 mouse model

**Fig. 4:**
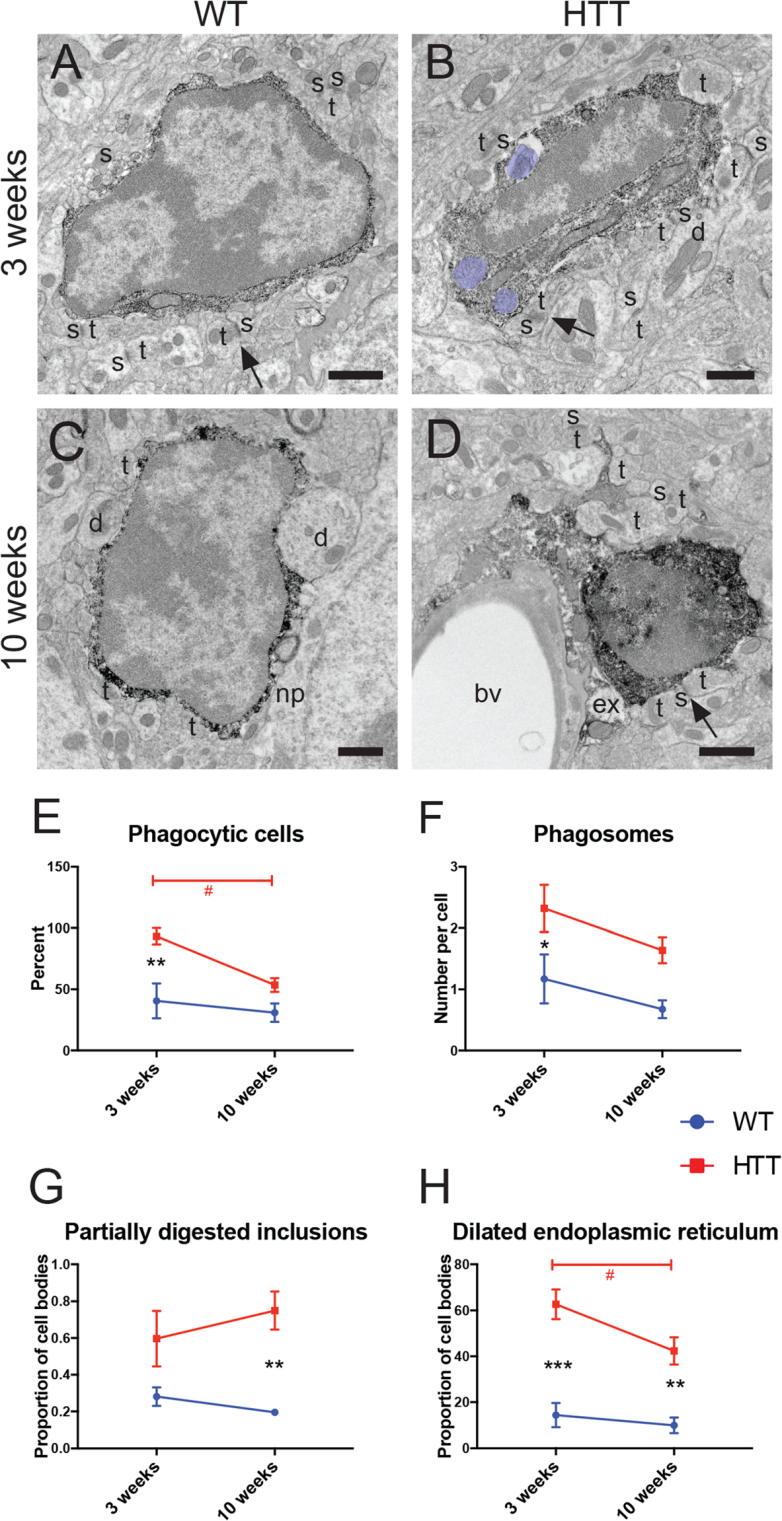
Altered microglial cell body ultrastructure observed in dorsomedial striatum during HD pathology. Electron micrograph examples of microglial cell bodies from 3-week-old WT (**A**), 3-week-old R6/2 (**B**), 10-week-old WT (**C**), and 10-week-old R6/2 (**D**) mice displaying IBA1+ immunoreactivity. Scale bar = 1 μm. The percentage of microglial cell bodies containing phagosomes (**E**) and the number of phagosomes per microglial cell body (**F**) were determined. The number of partially digested inclusions per cell body (**G**), as well as percentage of cells containing dilated endoplasmic reticulum (**H**), were measured. bv, blood vessel; d, dendrite; ex, extracellular digestion; np, neuronal perikaryon; s, dendritic spine; t, axon terminal. Black arrows point to excitatory synaptic clefts. Phagosomes are pseudocolored in purple. *n* = 7–11 cell bodies per animal for all conditions, and data was collected from *N* = 3–4 animals per condition. Asterisk denotes the difference from WT, red number sign denotes the difference between ages in R6/2 mice; *^,#^*p* < 0.05, **p < 0.01, ***p < 0.001

To further investigate the types of phagocytic cargo associated with the CD68+ puncta, we performed immunoEM in the dorsomedial striatum of 3-week- versus 10-week-old WT and R6/2 animals. We focused on the dorsomedial region of the striatum, which is one of the earliest regions affected by HD pathology [12]. Microglial cell bodies in both WT and R6/2 mice displayed characteristic ultrastructural features, including a cheetah-like heterochromatin pattern in their ovoid nuclei surrounded by a narrow band of IBA1+ cytoplasm (Fig. 4a–d). Microglial cell bodies were often found directly juxtaposed with neuronal elements such as cell bodies and dendrites, as well as synaptic elements, including axon terminals and dendritic spines (Fig. 4a–d). While microglia in both 3-week- and 10-week-old WT mice rarely showed processes contiguous with their cell body in ultrathin sections, microglia in 10-week-old R6/2 mice often had long, ramified processes connected to their soma in ultrathin sections (Fig. 4d). Microglial cell bodies across all experimental groups also displayed characteristic long stretches of ER and occasional lipidic inclusions and lipofuscin granules as well as lysosomes, all common in microglial cell bodies. A higher percent of microglia in R6/2 animals contained phagosomes (Fig. 4e), and microglia from R6/2 mice had more phagosomes per cell body than WT animals at 3 weeks (Fig. 4f), consistent with our light microscopy observations. Microglial phagosomes often held partially digested material, and microglia in R6/2 animals contained more partially digested inclusions than WT animals at 10 weeks (Fig. 4g), indicative of a possible impairment in phagolysosomal maturation.

In addition to these alterations, microglia in R6/2 striatum displayed increased frequency of dilated ER (Fig. 4h). Dilated ER is a well-known marker of cellular stress that has been described using EM in numerous contexts of neurodegeneration including amyotrophic lateral sclerosis and Alzheimer’s disease pathology [44, 56, 57]. We also identified two microglia in 3-week-old R6/2 mice with reduced IBA1 immunoreactivity and a condensed cytoplasm as well as nucleoplasm (Supplemental Figure 1). These cells are reminiscent of the dark microglia seen in aging and other neurodegenerative disease models [44, 57]. Their long processes formed acute angles and interacted with synaptic structures as well as the vasculature, all characteristic of dark microglia. However, we did not identify cells with the hallmark loss of nuclear chromatin pattern typically associated with dark microglia [57].

### Microglial process ultrastructure is altered in the R6/2 mouse model

**Fig. 5:**
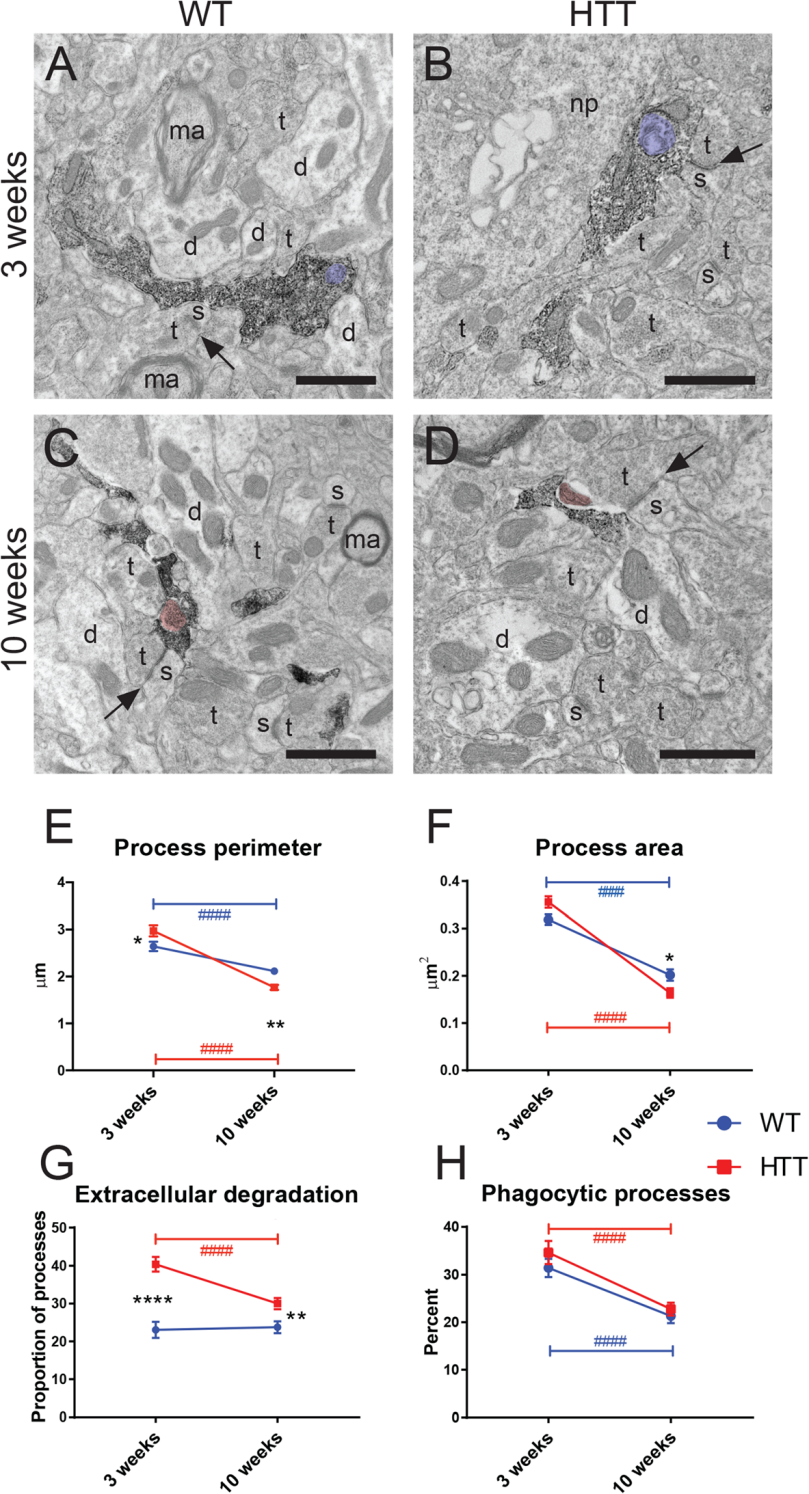
Altered microglial process ultrastructure observed in dorsomedial striatum during HD pathology. Electron micrograph examples of microglial processes from 3-week-old WT (**A**) and R6/2 (**B**) mice, as well as 10-week-old WT (**C**) and R6/2 (**D**) mice displaying IBA1+ immunoreactivity. The perimeter (**E**) and area (**F**) of microglial processes were calculated. The percentages of microglial processes surrounded by pockets of extracellular space containing degraded elements or debris (termed “extracellular degradation”; G) or containing phagocytic endosomes (**H**) was determined. Annotations are as follows: d, dendrite; ma, myelinated axon; np, neuronal perikaryon; s, dendritic spine; t, axon terminal. Black arrows point to excitatory synaptic clefts. Extracellular degradation is pseudocolored in pink; phagosomes are pseudocolored in purple. Scale bar = 500 nm, *n* = 70–100 microglial processes per animal for all conditions, *N* = 3–4 animals per condition. Asterisk denotes the difference from WT, blue number sign denotes the difference between ages in WT mice, red number sign denotes the difference between ages in R6/2 mice; *p < 0.05, **p < 0.01, ****^,####^*p* < 0.0001

To complement microglial cell body ultrastructure, we utilized immunoEM to glean information into microglial processes activities in the striatum. Microglial processes are IBA1+, allowing them to be investigated at ultra-high resolution in EM (Fig. 5a–d). They are not usually contiguous with their cell body in ultrathin sections and form a variety of shapes and sizes as they move throughout the neuropil and survey their environment. Similarly to cell bodies, processes often contained phagocytosed material (Fig. 5a, b) and made frequent direct contacts with extracellular degraded elements or debris (referred to as “extracellular degradation,” Fig. 5c, d). Microglial processes observed in WT and R6/2 mice displayed age-dependent decreases in their perimeter (Fig. 5e), suggesting that microglia are taking on a more surveillant morphology as indicated by our light microscopy studies. Interestingly, R6/2 microglial processes had larger perimeters than WT processes at 3 weeks, but their process perimeter was significantly reduced with age and became smaller than WT processes at 10 weeks (Fig. 5e). Microglial processes in both WT and R6/2 striatum reduced their areas with age, but again, microglial processes in R6/2 mice became significantly smaller in area than those of WT mice by 10 weeks (Fig. 5f). Microglial processes in R6/2 mice were also more likely to perform extracellular degradation than processes in WT animals, although this phenomenon also decreased with age (Fig. 5g). Both WT and R6/2 processes displayed an age-dependent decrease in phagocytosed material (Fig. 5h). Interestingly, this is in contrast with our findings for cell bodies indicating there may be a shift in phagocytic cargo trafficking between processes and cell bodies with maturation.

### Microglial processes make less contacts with synapses in the R6/2 mouse model

**Fig. 6:**
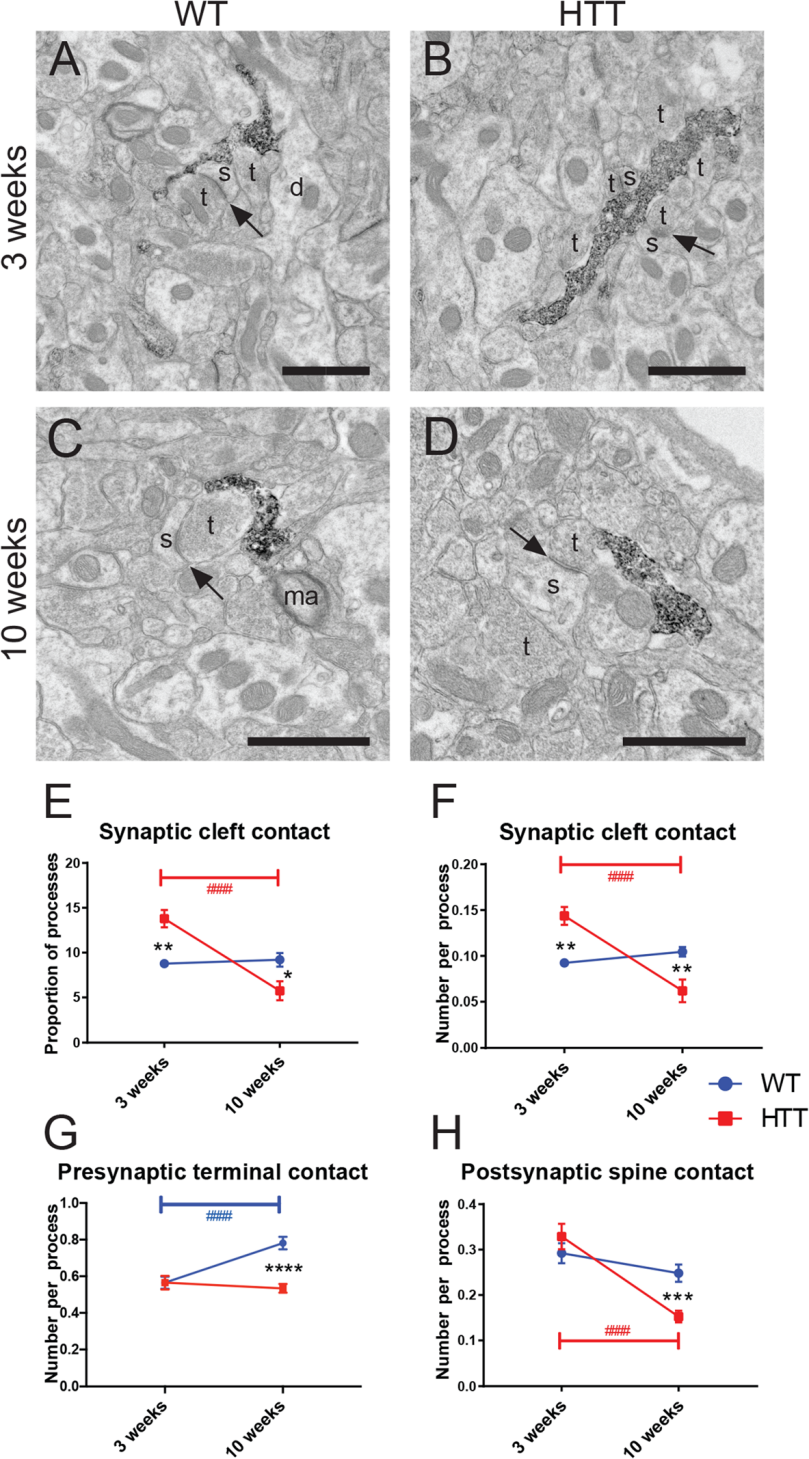
Microglia-synapse interactions in the dorsomedial striatum during HD pathology. Microglial processes from 3-week-old WT (**A**) and R6/2 (**B**), as well as 10-week-old WT (**C**) and R6/2 (**D**) mice displaying IBA1+ immunoreactivity and contacting synaptic structures. Proportion of microglial processes making contact with excitatory synaptic clefts (**E**) as well as the number of synaptic cleft contacts per process (**F**) was calculated. Microglial process interaction with presynaptic axon terminals (**G**) and postsynaptic dendritic spines (**H**) was also determined. Annotations are as follows: d, dendrite; ma, myelinated axon; s, dendritic spine; t, axon terminal. Black arrows denote excitatory synaptic clefts. Scale bar = 500 nm. *n* = 70–100 microglial processes per animal for all conditions, *N* = 3–4 animals per condition. Asterisk denotes the difference from WT, blue number sign denotes the difference between ages in WT mice, red number sign denotes difference between ages in R6/2 mice; *p < 0.05, **p < 0.01, ***p < 0.001, ****^,####^*p* < 0.0001

Some of the most striking changes in microglial process ultrastructure in R6/2 animals were their interactions with synaptic structures. We found many incidences of microglial processes interacting with synaptic structures, including axonal terminals, dendritic spines, and direct contact with excitatory synaptic clefts, defined as the junction between a presynaptic axon terminal and a postsynaptic dendritic spine (Fig. 6a–d). Microglia in WT dorsomedial striatum consistently interacted with the same number of excitatory synapses onto dendritic spines regardless of age investigated (Fig. 6e, f). However, microglia in R6/2 dorsomedial striatum displayed an age-related decrease in synaptic interactions (Fig. 6e, f). Microglial processes in R6/2 mice were also shifted from being more likely to interact with synaptic clefts than WT at 3 weeks of age to less likely at 10 weeks of age (Fig. 6e, f).

### Synaptic density does not increase with maturation in the R6/2 mouse model

**Fig. 7:**
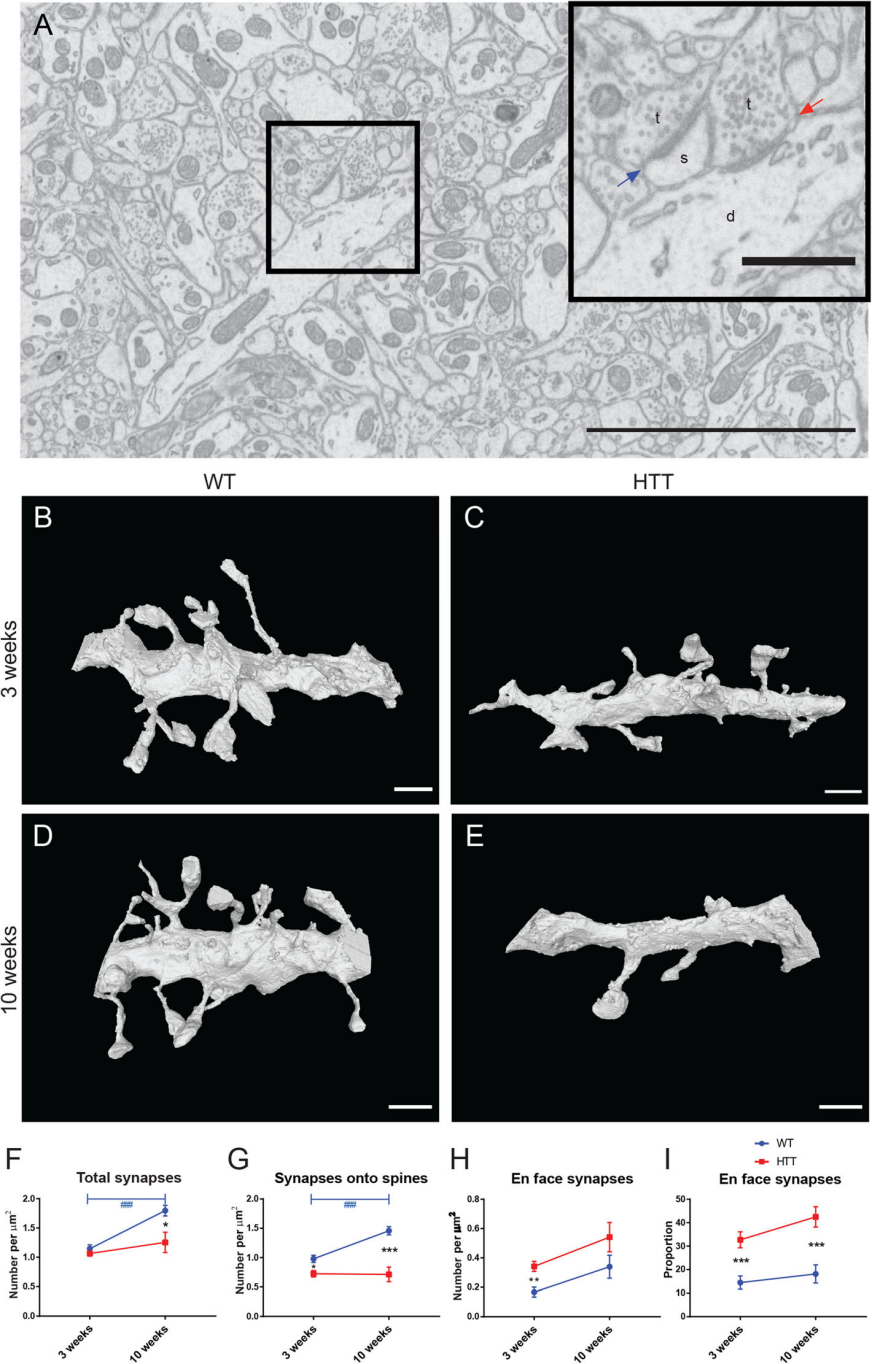
Synaptic density in dorsomedial striatum during HD pathology. A single image from the 125-μm^3^ 3-dimensional FIB-SEM stack (**A**) displays dense neuropil containing many dendrites, dendritic spines, and axon terminals. The dendrite (d) in the insert displays an en face synaptic contact from an axon terminal (t, red arrow) and a postsynaptic dendritic spine (s) directly contacted by an axon terminal (blue arrow). FIB-SEM was performed to create 125-μm^3^ images from 3-week-old WT (**B**) and R6/2 (**C**) and 10-week-old WT (**D**) and R6/2 (**E**) mice. Dendrites were traced using Ilastik and rendered using meshlab software. The number of synapses was calculated for each dendrite and normalized to their length (**F**). The number of synapses onto dendritic spines (**G**) and directly made onto the dendrite itself (**H**) was calculated and normalized to dendritic length. The proportion of en face synapses was calculated for each dendrite and averaged (**I**). Scale bar = 5 μm in **A**, 1 μm in insert, 1 μm in **B**–**E**. *n* = 3–6 dendrites per animal, *N* = 3–4 animals per condition. Asterisk denotes difference from WT, blue number sign denotes difference between ages in WT mice; *p < 0.05, **p < 0.01, ***^,###^*p* < 0.001

Microglia-synapse interactions may have an impact on synaptic density and could be impacted by a large number of factors including synaptic number. In order to investigate these changes, we analyzed dendrites in the dorsomedial striatum of 3-week-old and 10-week-old animals. We performed FIB-SEM experiments to image 150-250 μm^3^ synaptically dense regions—outside of striosomes and containing no blood vessels, myelinated axons, or cell bodies (Fig. 7a). Afterwards, we segmented randomly selected dendrites of lengths varying between 4.5 and 10 μm, dependent upon their orientation through the imaged volume (Fig. 7b–e). We correlated the segmented dendrite with the original images to count the number of excitatory synapses and determine the number of synapses per micrometer of dendrite (Fig. 7f). This analysis revealed that synaptic density was not affected by genotype in 3-week-old animals. However, synaptic density increased between 3 and 10  weeks in WT animals, without concomitant increase of synaptic density in R6/2 animals (Fig. 7f). This data supports other studies finding impairment of corticostriatal communication in 10–12-week–old R6/2 mice [58] and could be related either to synaptic loss or a defect of synapse formation or maturation.

### Synapses preferentially target dendritic trunks versus spines in the R6/2 mouse model

Because we segmented dendrites and counted synaptic density at nanoscale resolution, we were also able to discriminate between non-synaptic (spines which were not juxtaposed with an axon terminal containing synaptic vesicles) and synaptic spines (spines directly juxtaposed with an axon terminal containing synaptic vesicles). We were also able to count en face synapses formed directly onto the dendrite trunk (Fig. 7a, inset and Supplemental Video 1). Although there was no change in synaptic number at 3 weeks of age (Fig. 7f), synapses in 3-week-old R6/2 animals were significantly less likely to contact spines and did not increase synapses onto dendritic spines with age (Fig. 7G). Synapses in R6/2 animals were more likely to directly target the dendritic trunk itself compared to WT animals at 3 weeks of age (Fig. 7h). These data indicate that, while total synaptic density may not be affected in 3-week-old R6/2 animals, there is already a difference in the type of synaptic input made onto the medium-sized spiny neurons in the dorsomedial striatum. This shift in synaptic location (higher proportion of en face synapses to spine synapses) persisted in 10-week-old R6/2 animals (Fig. 7i).

## Discussion

We investigated microglia in the R6/2 mouse model at early, mid, and late disease stages using a combination of light and state-of-the-art ultrastructural analyses. Microglial density was higher in the striatum of R6/2 mice compared with WT mice at all ages investigated and significantly decreased with age in R6/2 mice. This is in line with prior studies finding that microglial brain density decreases in mice from 3 to 6 weeks of age, at which point it stabilizes [59]. Interestingly, we also found that microglia from 3-week-old WT mice had an increased NND, implying more distance between individual cells. There were no overt changes in cell body area between WT and R6/2 mice, although microglial cell body area decreased significantly between 3 and 13 weeks of age in R6/2 animals. We also noted a decreased morphological index (ratio of cell body to arborization area) in 3-week-old R6/2 mice versus WT littermates. These densitometric and morphological changes are all consistent with a more mature microglial phenotype [33], indicating long processes surveilling large areas of neuropil and monitoring synaptic activity in 3-week-old R6/2 mice.

Increased microglial process arborization may indicate increased synaptic interactions with the still-healthy MSSNs which make up 95% of the neurons residing in the striatum. Ma and colleagues described microglial morphological changes occurring as early as 7 weeks of age in R6/2 mice; however, our work provides the first quantification of microglial morphological changes in this model, and we focused on 3-week-old animals as well as animals with established behavioral deficits [60]. These overall data raise the intriguing possibility that microglial function may differ in R6/2 mice prior to the development of any motor impairments or synaptic loss, which are known to emerge at 6–7 weeks of age in this model [16, 35]. Recent single-cell mass cytometry (CyTOF) studies of microglia isolated from the whole brain of R6/2 mice identified three populations of microglia, including disease-associated cells found only in R6/2 mice. The disease associated cells were present in all ages investigated (4, 7, 10, and 13 weeks of age), but did not increase in number as the disease progressed [61]. Our data also showed age-dependent decrease in microglial density in the striatum of R6/2 mice at all investigated ages. In 4-, 7-, and 10-week-old R6/2 mice, the disease associated cells displayed a high expression of canonically anti-inflammatory cytokine IL-10 [61]. This is in line with our morphological analysis of microglia in 3-week- and 10-week-old animals which defined a surveillant, phagocytic, but not proinflammatory, phenotype. Further investigation into microglial phenotype, especially at early ages, is warranted in R6/2 and other mouse models of HD.

One of the unexplained aspects of HD lies in the functional changes that occur in microglia in presymptomatic and symptomatic carriers of mHTT. Changes in microglial metabolism are apparent in presymptomatic human carriers of mutant HTT, as visualized by positron emission tomography imaging [28, 29], and microglia in mouse models of HD express higher levels of phagocytic genes including those from the complement family [25, 27]. While microglia express increased phagocytic receptors, no changes in synaptic marker levels are apparent in the R6/2 mouse model until at least 6 weeks of age [16]. Our data has found increased microglial phagocytosis in the dorsomedial striatum in 3-week-old R6/2 mice, prior to the model’s development of overt neurological phenotypes or changes in synaptic markers. Our ultrastructural studies also uncovered increased microglial-synaptic interactions in the dorsomedial striatum of 3-week-old R6/2 mice. These data may point to an early role of microglia in the loss of synaptic input into the striatum seen in HD pathogenesis. The loss of synapses seen in later ages of R6/2 animals could be caused by alterations in the formation or maturation of synapses, synaptic loss, or by excess pruning of synapses by microglia. It is also possible that the microglial-synaptic interactions altered in R6/2 animals are associated with the instability of dendritic spines in this model [62]. Dendritic spines in R6/2 mice were shown to be less stable than those of WT animals as early as 5 weeks of age [62], although further research will be required to determine if this is causative or resulting from the changed microglial interaction seen here as early as 3 weeks and that also persisted at 10 weeks.

Our use of 3D EM to investigate synaptic structure and density revealed early alterations in synaptic location in presymptomatic HD mouse model. This technique could also be useful to investigate a number of other pathologies. It has been well described that synaptic loss precedes much of the cognitive impairment seen in Alzheimer’s disease patients and animal models of the disease [63]. In fact, recent 3D FIB-SEM studies have uncovered alterations in synaptic structure in human tissue in regions both near and far from amyloid plaques [64, 65]. Synapses in the transentorhinal cortices of AD individuals were more likely to target dendritic trunks, similar to our data in the striatum of the R6/2 model of HD [65]. Further research on larger datasets is required to determine if this shift in synaptic placement occurs across other brain regions and in other neurodegenerative diseases. Other opportunities available due to this technique involve determining if presynaptic terminals or postsynaptic spines change size or morphology in R6/2 mice, and are the focus of ongoing studies.

Microglia-specific expression of mHTT causes increases in proinflammatory signaling and exaggerated response (or priming) to sterile inflammatory factors [27]. Interestingly, the changes in microglial responsiveness in the model expressing mHTT in microglia alone occur during early adulthood (8 weeks of age), significantly before the neurological deficits are identified in mice expressing mutant HTT in astrocytes or oligodendrocytes using cell-type-specific promoters [18, 19]. Striatal microglia in presymptomatic R6/2 mice also contained increased levels of ferritin, which remained elevated throughout disease development [66]. While several studies underline the possibility that mHTT expression causes microglial-autonomous impairments, a recent study found microglial expression of mHTT was insufficient to cause HD symptoms, and that removing mHTT from microglia specifically did not ameliorate HD-associated features [67]. However, microglial phenotype (density, morphology, phagocytosis, etc.) was not specifically investigated. It is also important to note that while the R6/2 mouse model uses the endogenous HTT promoter and microglia express mHTT RNA, there have been no direct observations of HTT inclusions within microglial cell bodies [68, 69]. It is possible that microglia play large roles in HD pathogenesis even without expressing mHTT, and that most of the microglial alterations seen in our studies are a result of microglial responses to impaired neuronal function.

In addition to microglia, recent research has uncovered potential cell-type specific roles of various glial cells in HD. Expression of mHTT specifically in either astrocytes or oligodendrocytes causes neurological deficits, impaired motor functions, and early death [18, 19]. Researchers engrafted Rag1 null mice with human glial progenitor cells expressing normal (18Q) or mHTT (48Q) and found impaired coordination as evidenced by latency to fall from the rotarod, and their striatal neurons were hyperexcitable. Conversely, R6/2 mice injected with normal HTT expressing human glial progenitor cells displayed increased motor skills and longer survival than mice injected with R6/2 expressing human glial progenitor cells [70]. These studies draw attention to the importance of studying microglial interactions with other cell types expressing mHTT in the pathogenesis of HD. Overall, these data indicate that microglia may play an intimate role in the development and pathogenesis of HD pathology. Given that microglial alterations occur as early as 3 weeks of age, microglia remain a promising target for early disease therapeutic intervention. Further studies on microglial cytokine expression and transcriptome alterations are warranted to determine if pharmacological intervention to shift microglial phenotype could affect disease pathogenesis.

## Conclusion

In our study, striatal microglia displayed significant differences in density, distribution, morphology, phagocytosis, ultrastructure, and synaptic interactions, before any previously reported neuronal loss or behavioral deficits in the R6/2 mouse model. These alterations observed during HD pathology occurred concurrent with the synaptic alterations we describe at nanoscale resolution. Considering these findings with previously obtained information about the changes in inflammatory cells, both in the brain and periphery of human cases and animal models of HD, it is apparent that further studies into the potential role of microglia in HD are warranted.

## Supplementary information

**Additional file 1: Figure S1.** Stressed microglia in dorsomedial striatum of 3-week-old R6/2 mice. A microglia with condensed cytoplasm (A, inset in B) but normal heterochromatin (hc) patterning. The cell body is lightly IBA1+ and contains dilated ER (er, identified by white arrow). Another stressed microglia with condensed cytoplasm (C) displaying dilated ER (white arrow) occupies a satellite position next to a neuronal perikaryon (np) and shows an invaginated nucleus (identified by black arrow). It is also contacted by IBA1+ microglial processes. Scale bar = 1 μm. bv: blood vessel, ma: myelinated axon, mt: mitochondrion, s: dendritic spine, t: axon terminal.

**Additional file 2: Video S1.** Segmentation and 3D rendering of dendritic spine versus *en face* synapses. A dendrite from the dorsomedial striatum of a 3-week-old WT mouse is rendered in blue. A segmented axon (orange) makes a synapse directly onto a dendritic spine. A separate segmented axon (yellow) makes a dynapse directly onto the dendritic trunk.

## Data Availability

The datasets generated and analyzed during the current study are available from the corresponding authors on reasonable request.

## Abbreviations

3D: 3-dimensions
AD: Alzheimer’s disease
CyTOF: Single-cell mass cytometry
DAB: Diaminobenzidine
DAPI: 4′,6-diamidino-2-phenylindole
EM: Electron microscopy
ER: Endoplasmic reticulum
FIB-SEM: Focused ion beam scanning electron microscopy
HD: Huntington’s disease
HTT: Huntingtin gene
IBA1: Ionized calcium binding adaptor molecule 1
immunoEM: Immunoperoxidase staining for electron microscopy
mHTT: Mutant huntingtin
MSSN: Medium size spiny neuron
NaBH_4_: Sodium borohydrate
NND: Nearest neighbor distance
PBS: Phosphate-buffered saline
PET: Positron emission tomography
PFA: Paraformaldehyde
TB: Tris buffer
TBS: Tris-buffered saline
TEM: Transmission electron microscopy
TSPO: Translocator protein
WT: Wild-type
