# Supplementary figures and images for: Microglial physiological properties and interactions with synapses are altered at presymptomatic stages in a mouse model of Huntington’s disease pathology
Source: J Neuroinflammation. 2020 Apr 2;17:98. doi: 10.1186/s12974-020-01782-9 (PMC7118932; doi:10.1186/s12974-020-01782-9)

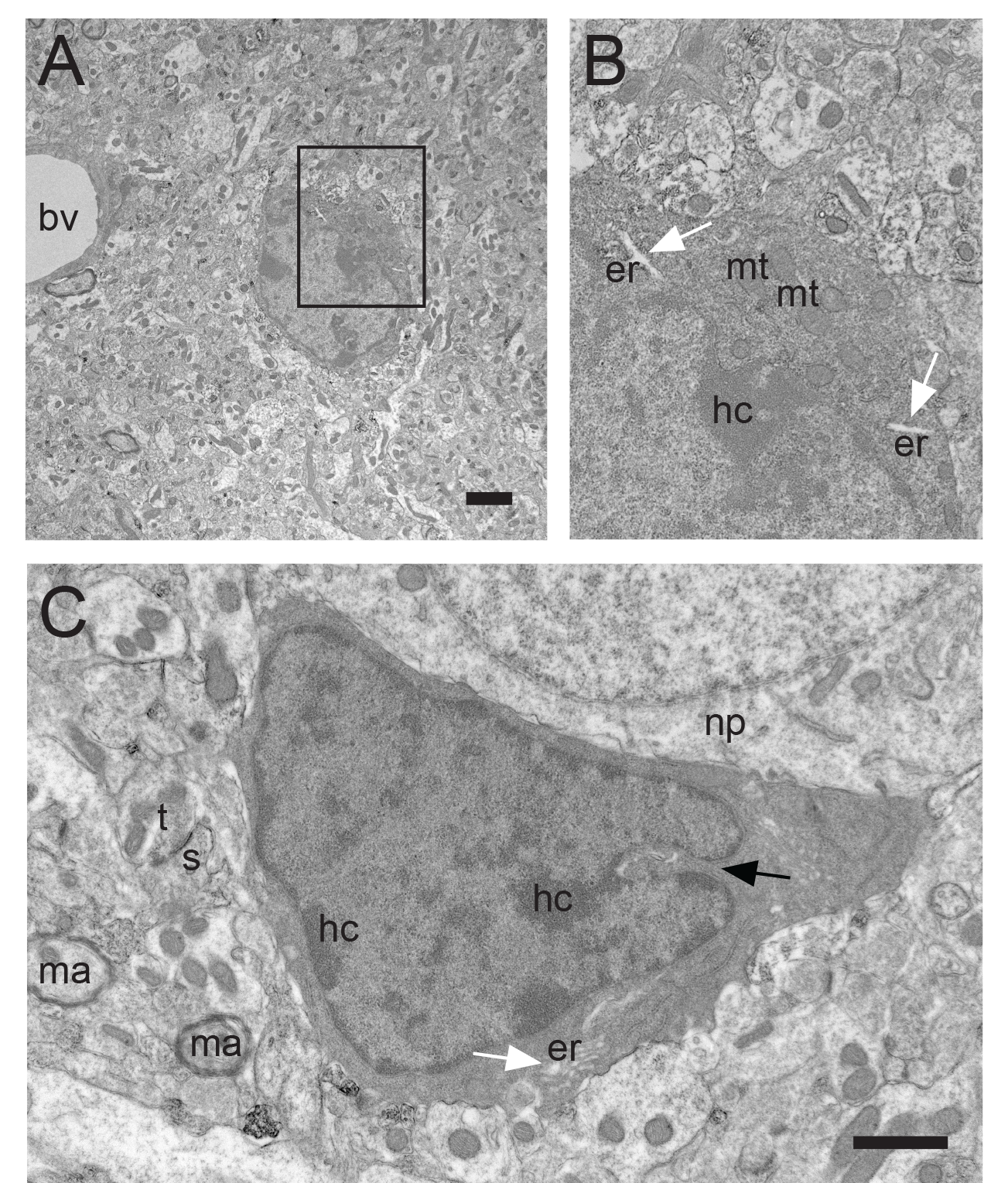

Supplement: Supplementary file 1 — Additional file 1: Figure S1. Stressed microglia in dorsomedial striatum of 3-week-old R6/2 mice. A microglia with condensed cytoplasm (A, inset in B) but normal heterochromatin (hc) patterning. The cell body is lightly IBA1+ and contains dilated ER (er, identified by white arrow). Another stressed microglia with condensed cytoplasm (C) displaying dilated ER (white arrow) occupies a satellite position next to a neuronal perikaryon (np) and shows an invaginated nucleus (identified by black arrow). It is also contacted by IBA1+ microglial processes. Scale bar = 1 μm. bv: blood vessel, ma: myelinated axon, mt: mitochondrion, s: dendritic spine, t: axon terminal. [file 12974_2020_1782_MOESM1_ESM.tif]
